# Development and implementation of a workshop for young adults with diabetes entering college and the workforce

**DOI:** 10.3389/fendo.2023.1288215

**Published:** 2023-10-10

**Authors:** Margaret West, Aniket Sidhaye, Meredith Thivierge, Risa M. Wolf

**Affiliations:** ^1^ Department of Pediatrics, Division of Endocrinology, Johns Hopkins University School of Medicine, Baltimore, MD, United States; ^2^ Department of Medicine, Division of Endocrinology, Johns Hopkins University School of Medicine, Baltimore, MD, United States; ^3^ Center for Diabetes and Endocrinology, University of Maryland School of Medicine, Baltimore, MD, United States

**Keywords:** type 1 diabetes, continuous glucose monitors, prescriptions, transition, young adult

## Abstract

The process of transitioning from pediatric to adult diabetes care for adolescents and young adults is challenging. This transition period may include many life changes, and can be fraught with worsening glycemic control leading to increased risk for diabetes-related hospitalizations and complications. Research has demonstrated that increased support during this period can help maintain engagement in diabetes care. Transition guidelines highlight the importance of preparation and readiness for transition. In this article, we discuss the development, implementation and content of a workshop for patients and parents/caregivers preparing for the transition to college, the workforce and adult diabetes care.

## Background

The process of transitioning from pediatric to adult health care for adolescents and young adults with chronic conditions is challenging. This period is a time of major changes in school, work and living environment. Young adults with type 1 diabetes face even greater challenges during this transition period with the additional stress of managing their disease and maintaining glycemic control. Almost 50% of young adults report difficulties in transitioning from pediatric to adult diabetes care, and almost 30% disengage from medical care during this period (1–3). Attendance at follow-up appointments declines and there is often an associated rise in hemoglobin A1c levels (4, 5). In general, hemoglobin A1c levels during this age group are at their peak, and thus there is increased risk for hospitalization and diabetes-related complications (5, 6).

Studies have identified the need for more support during the transition period for young adults with type 1 diabetes (7). Trials implementing care navigators and intensive transition coordinator support have shown promising results in facilitating engagement and clinic attendance in this age group (8, 9). The American Diabetes Association (ADA) and others have developed transition guidelines to help guide providers during this period (10–12). The importance of transition preparation through education and counseling promotes transition readiness for both patients and their caregivers (11).

### Development of the diabetes transition/off to college event

In 2018, we developed a workshop for young adults and their parents/caregivers to help prepare them for transitioning into the workforce, moving out of their parents’ home, or going to college. We hosted this event in collaboration with key stakeholders including the JDRF and The DiabetesLink (formerly College Diabetes Network (CDN)). (**Appendix A**: Event flyer). Workshop invitations were directed to high school juniors and seniors, as well as young adults who recently graduated from high school or were entering the workforce, with both type 1 and type 2 diabetes. At our institution, patients are usually transitioned to adult diabetes care one-year after high school, but can remain in the pediatric practice at the discretion of the provider based upon transition readiness.

#### Event structure

The workshop was structured as a 2-hour event where the first hour included a series of brief presentations hosted by faculty, followed by a panel discussion featuring students who already transitioned to college or the workforce, and some with their parents/caregivers as dyads. (**Appendix B**: Agenda) The platform for this event was initially an in-person workshop, but due to the COVID-19 pandemic beginning in 2020, the workshop was switched to a virtual (zoom) platform. Both formats have been successful and engaging, but an in-person workshop allows for either small group discussions (one for the students and one for the parents), or panel discussions; whereas on the zoom platform a panel discussion has been easier to facilitate. In order to engage participants in the workshop, the hosts prepared a list of questions to get the panel started, and then the attendants were requested to submit questions to the host, which were then asked of the panel discussants. At the completion of the workshop, attendees were able to provide feedback through a brief survey of what was most helpful and what could be improved in the program.

#### Event timing

The first year that we hosted this event in 2018, the event was held on a Sunday in May, but feedback from parents was that it felt too close to the time the students were leaving for college or moving out of the house and felt that more preparation time would have been helpful. Based on this feedback, in subsequent years, this event has been held on a Sunday in March.

### Topics covered during the workshop

#### Finding an adult diabetes provider

This presentation is given by an adult endocrinologist. During this portion of the presentation, we emphasize to patients and their parents that the overall goals of care are similar between adult and pediatric providers, namely, to help the patient achieve the best control of blood glucose that is possible for them, and to ensure good health now and in the future. The adult provider’s perspective includes more focus on control of cardiovascular risk factors and management of microvascular complications. However, unlike tertiary care pediatric centers, many adult providers do not have onsite nutritionists, psychologists and diabetes educators so often separate appointments for these providers have to be made. To ensure good continuity of care, the participants are reminded that they should identify potential adult providers 6-9 months ahead of time since adult clinics often have long waits for new patient appointments and that a good time for such an appointment could be soon after graduation from high school, or later depending on the planned transition time. At the first appointment with their new provider it’s important to identify key clinical and administrative staff in the office, as well as procedures for obtaining refills. If the patient is going away for college, we discuss that some individuals choose to have a second provider at their college, while maintaining a provider in their hometown. Regardless of diabetes provider location, we recommend setting up an appointment with the college student health center for the student to be aware of local resources for diabetes care and management.

#### Setting up disability accommodations at college

This presentation discusses the process of registering at their school’s Disability Services office prior to the start of classes to facilitate reasonable accommodations for diabetes management in the classroom and on campus. While some students are hesitant to self-identify as having a disability, and are comfortable with informal accommodations provided in high school, they are encouraged to register with Disability Services prior to an issue arising to ensure equal opportunity to succeed in college. Students are encouraged to share a list of proposed accommodations and rationales with their medical team for submission. In this presentation, we review commonly requested accommodations such as ability to pause exams in case of hypoglycemia or hyperglycemia, continuous access to diabetes technology including cell phones for continuous glucose monitoring, and permission to keep a minifridge in the dorm room for insulin storage. As an example, the speaker will demonstrate how to navigate a local college website to identify the Disability services office and find the appropriate forms and contact information.

#### Establishing care and obtaining prescriptions at college

##### Maintaining diabetes care during college

This presentation encourages students to maintain frequent communication with their primary pediatric or adult diabetes team while in college, and have their team’s contact information readily accessible for urgent or after-hours needs. We also suggest students identify the nearest emergency room in case of an emergency, and also determine the capabilities and limitations of the student health office if unable to reach their primary endocrinology team.

##### Prescription planning

Students and parents should also develop a plan for obtaining prescriptions and supplies, and whether these will be picked up locally or shipped from their parents’ home. Prescription plan logistics will depend on the student’s insurance as well as proximity to home. We highlight the importance of developing a system for the student to identify when to begin the reordering process to avoid running out of medications or supplies. Families are encouraged to transition the responsibility for medication and supply management to the student during senior year of high school so that parents can provide oversight and guidance before the student moves away from home. This is especially important given the frequent confusion on whether supplies are coming from a pharmacy or DME company, and who to contact for refills. Patients are also encouraged to identify a local pharmacy near the college in case of an urgent medication need.

#### Nutrition & physical activity

This presentation is given by a dietitian (RD), who discusses navigating the dining hall in college and adjusting to a variable eating schedule typical in college or in the workforce. Nutrition choices and food intake often change when young adults leave home and live at college. Dining halls and cafeterias can be challenging to navigate for both food choices and carbohydrate counting. During this presentation, the RD discusses strategies on carbohydrate counting in dining halls and college food establishments. Some colleges have nutrition information on their dining websites, or have phone apps available so that students can see menus and associated nutrition information. The speaker demonstrates how to navigate a school nutrition website for a local college as an example, and how to contact the campus dietitian for additional information. An email/letter template is shared for contacting the school dietitian with request for carbohydrate information and possibly food additions or modifications to the cafeteria menu (e.g. sugar-free pancake syrup, high protein yogurt).

College dining halls often have many food options for main courses, sides, and desserts. The RD discusses how to make healthy food choices for balanced meals, in order to avoid large glycemic excursions, promote overall heart healthy food intake and healthy weight management. Estimating portions is also reviewed as the college student is unlikely to use measuring cups in the dining hall. Sample serving sizes are visualized with images and objects for different food types to help students estimate carbohydrate content.

The speaker also discusses managing meals with a variable schedule of classes and activities in college, and managing late-night eating. The dietitian reviews tips such as planning meals and snacks, which may include packing food to go, or learning about different dining halls on campus and their hours of operation. The RD provides suggestions for complex carbohydrates and protein sources to keep in their dorm room, which can be eaten as a quick meal or on-the-go.

In the last part of the nutrition presentation, the dietitian discusses the benefits of physical activity and ways to stay physically active on campus while also managing blood glucose with exercise. The speaker reviews exercise management with multiple daily injection verses insulin pumps (including hybrid closed loop systems). The discussion includes utilizing exercise functions on pumps, modifying insulin doses at meals prior to exercise, and food choices to help maintain blood glucose levels during activity. The RD provides guidance on complex carbohydrate and protein consumption to help optimize athletic performance, with examples such as peanut butter sandwich on whole wheat bread, or protein granola bar. The speaker also discusses differing carbohydrate needs for blood glucose maintenance and treatment of hypoglycemia depending on type of insulin delivery (injections, pumps, and hybrid-closed loop systems).

#### Managing sick days, hyperglycemia and hypoglycemia

This presentation reviews hyperglycemia, sick day management and hypoglycemia management. This includes a review of when to check for ketones, managing hyperglycemia when sick, and the signs and symptoms of DKA. A list of scenarios of when to seek help are reviewed. Hypoglycemia management is also reviewed. Importantly, if students have roommates, we remind them to demonstrate to the roommate where the glucagon is stored and how to give glucagon in an emergency. The list of contents for an “emergency diabetes kit” are outlined. In this discussion, wearing a medical alert bracelet is reinforced, as well as setting up a Medical ID on the phone for emergency responders to access if needed.

#### Consumption of alcohol and other legal substances for young adults over age 21

This presentation reviews responsible behavior, and effects of alcohol and other substances on diabetes. While consumption of alcohol is not condoned, it is a likely exposure after high school or in college, and thus it is important to discuss the effects of alcohol on diabetes. This presentation reviews that alcohol is predominantly metabolized by the liver, and with alcohol consumption, the liver’s ability to perform gluconeogenesis and glycogenolysis is reduced and can lead to hypoglycemia. Thus, it is recommended to drink alcohol responsibly and limit to 1-2 drinks, always eat carbohydrates while drinking alcohol, and monitor blood sugars closely for potential delayed hypoglycemia (13).

In some states, recreational cannabis is legal for young adults 18 years of age and over. Recreational cannabis use can result in acute adverse events with increased risk for DKA and higher hemoglobin A1c. In this presentation, we review the effects of marijuana, and the increased food consumption typically associated with cannabis use that can lead to hyperglycemia.

#### Communication with colleagues, roommates and parents

##### Communication with peers and instructors

This presentation is usually given by a psychologist at our center, but can be given by anyone on the diabetes team. Upon arrival to college, students should share their diabetes diagnosis with their roommate, resident assistant, and other important adults such as coaches. For those joining the workforce, it is important to inform co-workers and bosses. While these individuals are not expected to participate in the student’s diabetes management, they should know how to identify an emergency and respond when the student with diabetes needs assistance. In this presentation, the students are encouraged to practice having these conversations, as these initial conversations can be a source of anxiety. As an example, the speaker may role-play how to open this conversation with a new roommate, or start a conversation about diabetes with their athletic coach. Further, advice on seeking mental health resources and peer support on campus is discussed.

##### Communication with parents/caregivers

Parents and students are also encouraged to discuss expectations regarding communication, and set up a schedule for touching base about diabetes management successes or issues. With the advent of continuous glucose monitoring, families must establish boundaries around outreach to their college students regarding blood glucose management. Some families identify blood sugar thresholds above or below which parents will contact the student and/or other individuals such as roommate or resident assistants, while some families opt not to use glucose sharing technology. Students are also encouraged to utilize other resources and apps that connect to their continuous glucose monitors and notify them of an untreated low. The speaker also reminds parents to avoid focusing every conversation around diabetes when speaking with their children at college.

##### Partner organization resources

The series of presentations (**Table 1**) concludes with an overview of resources available from diabetes organizations, including JDRF and DiabetesLink (formerly College Diabetes Network, CDN). These resources are shared with the participants by email after the event. 

**Table 1:** Diabetes transition and off to college workshop content.

Presentation Title	Content covered in each presentation
Welcome & Introduction	-Transition period from adolescence to adulthood -Challenges in the diabetes transition period -Importance of transition preparation and readiness
Finding an Adult Provider	-Differences in adult and pediatric diabetes care -Scheduling with an adult provider in advance
Nutrition & Exercise	-Navigating meals and eating on an erratic schedule -Considerations for carbohydrate counting in the dining hall -Planning for exercise and physical activity
Disability Services & Accessing Prescriptions	-Setting up accommodations with the Disability office -Considerations for accommodations at school and work -Transitioning management of prescriptions from caregiver to child -Prescription planning at college and identifying local pharmacies -Identifying local care for urgent issues and emergencies
Managing Sick Days, Hyper- and Hypoglycemia	-Review management for sick days and checking for ketones -Acute management of hypoglycemia review -Emergency medications and training roommates
Alcohol and Drugs with Diabetes*	-Responsible intake of alcohol and adverse effects on diabetes management -Marijuana use and effects on diabetes (in states where it is legal) *if applicable to students >21years of age
Healthy Communication	-Communications about diabetes with peers, professors and coaches -Managing communication expectations between students and parents/caregivers
Partner Organization Resources	-Invite local diabetes organizations to present and share resources (example: JDRF, The Diabetes Link)

## Discussion

Transition preparation and readiness are important to ensure a smooth transition process from pediatric to adult diabetes care that occurs during a time of many life-transitions. Readiness should apply to both adolescents and young adults, as well as their parents and caregivers (9). The implementation of a workshop to prepare and ready patients and caregivers for the upcoming transition to college or into the workforce, and leaving the home has been a successful addition to our transition program for all young adults with diabetes. Feedback from patients and caregivers has reflected important knowledge acquisition, and insight on how to set-up accommodations at college or work, eating on a variable schedule, and ideal communication patterns among all stakeholders. We hope this article provides a framework for other diabetes programs to establish and host a similar program for their young adults and patients transitioning to college or the work-force.

## Data availability statement

The original contributions presented in the study are included in the article/**Supplementary Material**. Further inquiries can be directed to the corresponding author.

## Author contributions

RW: Conceptualization, Project administration, Resources, Supervision, Visualization, Writing – original draft, Writing – review & editing. MW: Conceptualization, Project administration, Resources, Visualization, Writing – original draft, Writing – review & editing. AS: Conceptualization, Project administration, Resources, Writing – original draft, Writing – review & editing. MT: Conceptualization, Project administration, Resources, Visualization, Writing – original draft, Writing – review & editing.
