# Supplementary material for: Development and implementation of a workshop for young adults with diabetes entering college and the workforce
Source: Front Endocrinol (Lausanne). 2023 Oct 10;14:1288215. doi: 10.3389/fendo.2023.1288215 (PMC10598457; doi:10.3389/fendo.2023.1288215)
Supplement: Supplementary file 1 [file DataSheet_1.pdf]

Add orgnaization logo

Add orgnaization logo

Add orgnaization logo

Teen Transition Years and Off to College

The (*add name*) and (*add name of partner organization*) invite high school students living with diabetes and their caregivers/parents to this unique opportunity to learn about and discuss the many facets of the “teen transition years,” including going off to college, starting in the workforce, and transitioning from pediatric to adult diabetes care. If you currently have a child in college or are currently a student in college or preparing for adult medical care, you may also find this program valuable.

This program will give you the opportunity to hear from and ask questions of health care professionals who have experience in this field, as well as a panel of young adults living with diabetes who have first-hand experience with these transition years.

Sunday, March 26, 2023
10:00 am – 12:00 pm
Webinar held via Zoom

To register and obtain Meeting ID, please contact: *add name, email*

[redacted]
