# Supplementary material for: Development and implementation of a workshop for young adults with diabetes entering college and the workforce
Source: Front Endocrinol (Lausanne). 2023 Oct 10;14:1288215. doi: 10.3389/fendo.2023.1288215 (PMC10598457; doi:10.3389/fendo.2023.1288215)
Supplement: Supplementary file 2 [file DataSheet_2.pdf]

Organization logo Organization logo Organization logo

Off to College and the Teen Transition Years

Program Agenda

10am ***Welcome and Introductory Comments***
Speaker name; Affiliation

10:15am ***Focus Topic Presentations***

Finding an Adult Provider
Speaker name; Affiliation

Navigating the Dining Hall
Speaker name; Affiliation

Disability Services and Accessing Prescriptions
Speaker name; Affiliation

Managing Sick Days
Speaker name; Affiliation

Alcohol and Drugs with Diabetes
Speaker name; Affiliation

Healthy Communication
Speaker name; Affiliation

Partner Resources
Speaker name; Affiliation

11:15 am ***Panel and Q & A with parents and young adults***

11:55 am ***Wrap-up***
